# Rowell Syndrome in a Middle-Aged Woman: A Case Report

**DOI:** 10.7759/cureus.39631

**Published:** 2023-05-29

**Authors:** Diane Lee, Shayan Waseh, Kiran Motaparthi, Sylvia Hsu

**Affiliations:** 1 Dermatology, Temple University Hospital, Philadelphia, USA; 2 Dermatology, University of Florida, Gainesville, USA

**Keywords:** subacute cutaneous lupus erythematosus, sjogren's, targetoid lesion, erythema multiforme, lupus erythematosus, rowell syndrome

## Abstract

Rowell syndrome (RS) is characterized by the presentation of lupus erythematosus (LE) with erythema multiforme (EM)-like lesions. It is thought to display a characteristic serologic pattern consisting of a "speckled-type" antinuclear antibody (ANA), positive anti-Ro/SSA or anti-La/SSB, or positive rheumatoid factor (RF). We report the case of a patient with subacute cutaneous lupus erythematosus (SCLE) who presented with EM-like lesions responsive to oral corticosteroids.

## Introduction

Rowell syndrome (RS) was first defined as a distinct clinical entity by Rowell et al. in 1963. It is characterized by erythema multiforme (EM)-like lesions appearing in the setting of established lupus erythematosus (LE) and is found most commonly in middle-aged women [1,2]. It is thought to display a specific immunologic pattern consisting of "speckled-type" antinuclear antibody (ANA), positive anti-Ro/SSA or anti-La/SSB, and positive rheumatoid factor (RF) [1]. Around 95 cases of EM-like lesions associated with LE have been discussed in the literature [3]. We describe the case of a 51-year-old woman with previously diagnosed subacute cutaneous lupus erythematosus (SCLE) who presented with EM-like lesions consistent with the criteria of Rowell syndrome [1].

## Case presentation

**Figure 1:**
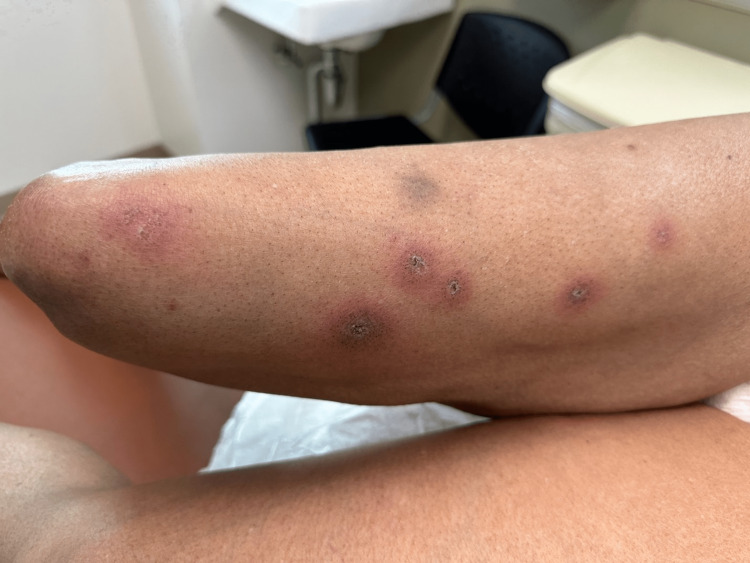
Dusky, targetoid lesions on bilateral thighs

**Figure 2:**
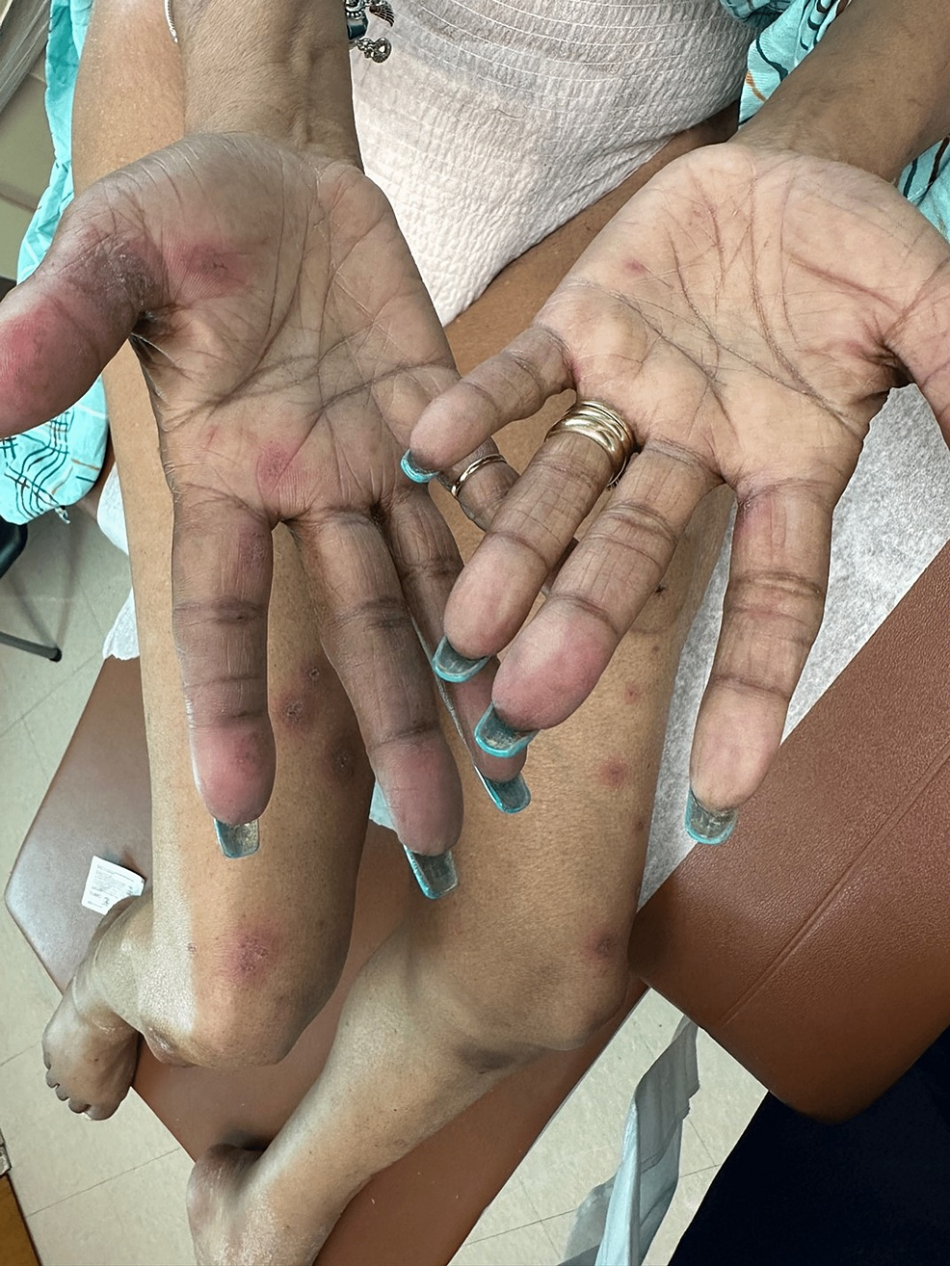
Similar lesions are seen on the upper and lower extremities. Note the dusky targetoid lesions on the bilateral palms, a characteristic location for erythema multiforme.

A 51-year-old woman presented with erythematous dusky patches on the arms, legs, and buttocks. She had a history of recently diagnosed SCLE and self-reported Sjogren’s disorder. She was started on clobetasol ointment two months prior to presentation for a dry, scaly rash thought to be a manifestation of SCLE. On physical examination, she was found to have round erythematous patches on her bilateral proximal upper and lower extremities (Figure 1) and her palms (Figure 2).

**Figure 3:**
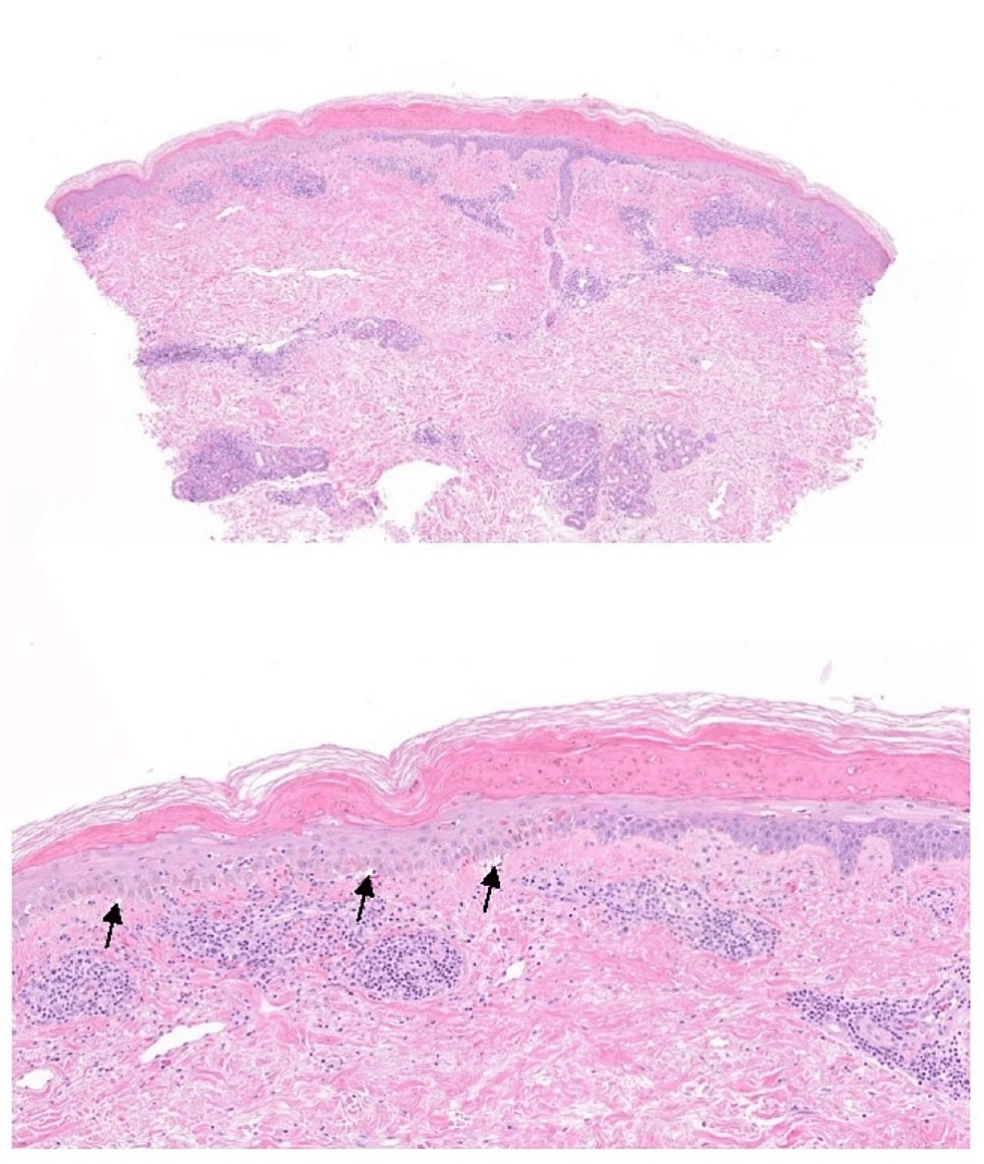
Top: biopsy of an EM-like lesion; bottom: black arrows pointing to vacuolar dermatitis

Two 4-mm punch biopsies of the right arm and left leg were obtained. Biopsy revealed vacuolar dermatitis with an atrophic epidermis and moderate periadnexal lymphocytic inflammation, consistent with lupus erythematosus (Figure 3).

The patient’s laboratory studies were positive for a markedly elevated Ro/SS-A (>8.0 AI, negative <1.0 AI).

The appearance of EM-like lesions in a patient with established lupus erythematosus and positive anti-Ro/SSA supported a diagnosis of RS. She was started on clobetasol 0.05% ointment BID and prednisone 40 mg daily, which was tapered over the course of approximately one month. The patient experienced resolution of her EM-like lesions with no recurrence upon discontinuation of prednisone.

## Discussion

Clinical presentation

RS was first described as a distinct clinical entity by Rowell et al. in 1963, who analyzed four patients with LE presenting with lesions resembling EM in the absence of identifiable EM triggers [1]. The patients had the specific serologic pattern of speckled-type antinuclear factor, precipitating antibody to saline extract of human tissue (anti-Ro/SSA), or rheumatoid factor [1,3]. Since its first description, various diagnostic criteria have been proposed for Rowell syndrome (Table 1). Fundamentally, Rowell syndrome is defined as LE with EM-like lesions in the presence of positive serologic markers such as speckled-pattern ANA, anti-Ro/SSA, or RF.

Histologically, RS is characterized by a dramatic vacuolar interface dermatitis that shares features of both EM and LE. While the presence of significant epidermal damage to the point of full-thickness epidermal necrosis is a shared characteristic with EM, the presence of periadnexal lymphocytic inflammation, parakeratosis, and/or periadnexal CD123 plasmacytoid dendritic cells help to highlight RS as an expression of LE [4].

Our patient’s presentation aligned with both the diagnostic and histopathologic criteria previously described for Rowell syndrome.

**Table 1:** Different diagnostic criteria for Rowell Syndrome

Rowell et al. (1963) [1]	Zeitouni et al. (2000) [5]	Torchia et al. (2012) [6]
LE; EM-like lesions (in the absence of precipitating factors); Serology: speckled pattern, ANA, anti-SJT (anti-Ro/SSA), positive RF	Major: LE; EM-like lesions (in the absence of precipitating factors); speckled pattern ANA	Major: CCLE; EM-like lesions; At least one of speckled ANA, anti-Ro/SSA, anti-La/SSB, negative DIF on EM-like lesions
	Minor: Chilblains; anti-Ro/SSA or anti-La/SSB, positive RF	Minor: Absence of infectious or pharmacologic triggers; absence of typical EM location (acral or mucosal); the presence of at least one additional ARA criteria for SLE, excluding discoid rash, ANA, photosensitivity, malar rash, and oral ulcers
All diagnostic criteria	All major, one minor	All major, at least one minor
LE: lupus erythematous; EM: erythema multiforme; ANA: antinuclear antibody; RF: rheumatoid factor; CCLE: chronic cutaneous lupus erythematous; DIF: direct immunofluorescence; ARA: American Rheumatism Association		

Pathogenesis

The underlying pathogenesis of Rowell syndrome has yet to be fully delineated. One of the current theories is that there is a type IV immune response, particularly in patients with anti-Ro antibodies. In the absence of classic exogenous antigens, Kim et al. propose that anti-Ro antibodies may trigger an EM-like pattern of type IV immune response along with antibody-dependent cellular cytotoxicity that causes typical lupus-like diathesis [7].

Management

Currently, RS is managed with a similar treatment regimen as LE, including immunomodulatory medications such as methotrexate and rituximab in refractory cases [8-11]. Most cases of RS are successfully managed with a combination of steroids, azathioprine, antimalarials, dapsone, or cyclosporine [2,3]. The length of treatment is variable. Our patient was successfully treated with one month of prednisone 40 mg daily, which resulted in rapid resolution of her lesions. No recurrence of her lesions was noted upon discontinuation of her prednisone taper.

## Conclusions

Rowell syndrome is a rare presentation distinguished by EM-like lesions in the presence of underlying LE and the absence of identifiable EM precipitants. It remains important to have high clinical suspicion for RS in patients with LE and EM-like lesions in order to treat patients appropriately and refine diagnostic parameters and pathophysiology further. Additionally, Rowell syndrome is increasingly being appreciated as an expression of LE with EM-like lesions rather than the co-occurrence of LE and EM. This case reaffirms and reinforces this modern understanding of RS.
